# The potential effectiveness of probiotics in reducing multiple sclerosis progression in preclinical and clinical studies: A worldwide systematic review and meta-analysis

**DOI:** 10.1371/journal.pone.0319755

**Published:** 2025-04-24

**Authors:** Zahra Zangeneh, Mosayeb Rostamian, Hamid Motamedi, Amirhooshang Alvandi, Ramin Abiri

**Affiliations:** 1 Fertility and Infertility Research Center, Health Technology Institute, Kermanshah University of Medical Sciences, Kermanshah, Iran; 2 Infectious Diseases Research Center, Health Institute, Kermanshah University of Medical Sciences, Kermanshah, Iran; 3 Asadabad School of Medical Sciences, Asadabad, Iran; 4 Medical Technology Research Center, Health Technology Institute, Kermanshah University of Medical Sciences, Kermanshah, Iran; University of Iowa Hospitals and Clinics Pathology: The University of Iowa Hospitals and Clinics Department of Pathology, United States of America

## Abstract

**Background and objective:**

Multiple Sclerosis (MS) is an immune-mediated disease characterized by nerve cell inflammation and demyelination. The effectiveness of probiotics in reducing inflammatory damage in MS. Therefore, the aim of this systematic review and meta-analysis was the potential effectiveness of probiotics in reducing Multiple Sclerosis progression in preclinical and clinical studies.

**Methods:**

PubMed, Scopus, Cochrane, and Google Scholar databases were searched using multiple relevant keywords, and screening was carried out based on the inclusion/exclusion criteria from January 2004 to August 16, 2024.

**Results:**

Based on our criteria, 269 papers were obtained, and after omission of unsuitable articles, 23 full-text articles consisting of 17 animal studies and six human models were selected. It was concluded that in an experimental autoimmune encephalomyelitis (EAE) animal model, probiotics such as *Bifidobacterium*, *Prevotella*, and *Lactobacillus* can decrease the T helper 1 (Th1)/Th17 ratio while inducing interferon gamma (IFN-γ) and interleukin (IL)-17 levels. In all cases, probiotics can modulate immune cells and cytokines and consequently decrease EAE signs and symptoms. In all human studies, single or multiple probiotics decreased the severity of disease and changed the gut microbiota population.

**Conclusion:**

Our results showed that probiotics can control the development of MS by reducing inflammatory conditions, and may have beneficial effects in the prevention and treatment of MS.

## Introduction

Multiple Sclerosis (MS) is an autoimmune disease characterized by autoreactive interferon gamma (IFN-γ) and interleukin (IL)-17 producing T cell-mediated inflammation, followed by neural cell demyelination [1]. Almost 2,500,000 individuals suffer from MS worldwide [2]. Although the pathogenesis of MS is multifactorial, it seems that inflammation and oxidative stress factors (OSF) are important causes of MS, and we believe that the severity of MS (according to the EDSS criteria) is directly related to the level of inflammation and OSFs [3,4]. Inflammation is caused by infiltration of immune cells and destruction of the myelin sheath. T and B lymphocytes can cross the blood-brain barrier (BBB), activate microglial astrocytes, and induce an inflammatory environment in which myelin is damaged and destroyed [5]. Owing to the similarity of clinical and pathophysiological features, the animal model of MS, which is called Experimental Autoimmune Encephalomyelitis (EAE), is an applicable archetype for studying the disease in non-human models [6]. EAE can be induced by immunization with myelin components and myelin protein peptides such as glycoprotein myelin oligodendrocyte (MOG) 35-55 [7].

Recently, several studies have highlighted the significance of the human microbiome in balancing and enhancing the immune system [8]. The gut-microbiota-brain axis describes a network of bidirectional communication between the gut and the CNS. This communication is crucial for regulating the homeostasis of the CNS and intestinal microbiota. Signaling through various chemical transmitters, neural mechanisms, and the immune system constitutes the communication pathways in these biological networks. The gut microbiota can influence the body through chemical communication, including both “direct” and “indirect” signals with the nervous system Short-chain fatty acids (SCFAs), such as butyrate, propionate, and acetate, are products of intestinal bacterial fermentation that regulate intestinal adaptive immune responses and play a vital role in CNS function. When the gut microbiome is disrupted, inflammatory cytokines and other immune cells are released, impacting the BBB and gastrointestinal permeability, as well as the levels of SCFAs and other metabolites that contribute to neuro-inflammation and demyelination [7]. Dysbiosis of the intestinal microbiota is implicated in several immune-mediated diseases, including inflammatory bowel disease, MS, type 1 diabetes, and rheumatoid arthritis [9]. Bacterial proliferation in the gut affects the immune system and can disrupt the gut barrier, resulting in an imbalance of T helper 1 (Th1)/Th2 responses, which leads to gastrointestinal autoimmune diseases [10].

Several diets have been studied in MS to modify the gut microbiome’s composition and reduce systemic inflammation, including the Swank diet, a low-fat regimen developed by Dr. R. L. Swank, and the Wahls diet, created by Dr. Terry Wahls, both of which have demonstrated beneficial effects in managing MS. Research indicates the effectiveness of probiotic-based therapies for treating MS through various mechanisms, often involving the reduction of demyelination and inflammation in the brain. It appears that greater emphasis should be placed on the significance of probiotics in the diets of these patients [11].

Probiotics can alter the gut microbiota and thus affect health status [11]. According to the World Health Organization (WHO), probiotics are living microorganisms that can improve the health benefits to the host“ [12]. In recent years, researchers have proposed probiotics as a new treatment option for autoimmune diseases [13].

Lactobacilli and bifidobacteria (predominant in the upper and lower parts of the intestine, respectively) show some therapeutic effects in experimental induced colitis, experimental inflammatory bowel disease, and experimental arthritis [14, 15]. For example, *Lactobacillus plantarum*, *Bifidobacterium lacti*s, *Lactobacillus acidophilus* and *Lactobacillus casei* have beneficial effects in colitis, food allergies, and arthritis models. However, little is known about the exact effects of lactic acid bacteria on MS/EAE.

Several studies have shown a relationship between intestinal microbiome dysbiosis and some gastrointestinal disorders in approximately 70% of MS patients [16]. Administration of probiotics or a mixture of probiotics, such as lactobacilli, streptococci, and bifidobacteria, can reduce the severity of EAE in mice, and there is evidence of the role of the gut microbiota in EAE development [17,18]. A recent study showed that Prevotella from the human gut suppresses EAE induction. Recently, it has been shown that changes in intestinal microbiota composition are associated with high levels of intestinal Th17 cells and enhanced disease activity in MS [19]. These findings indicate that the use of probiotics can be effective in patients with MS.

There is little information regarding the effects of probiotic bacteria on the central nervous system; therefore, the aim of this systematic review and meta-analysis was to evaluate the potential effectiveness of probiotics on MS in preclinical and clinical studies.

## Methods

### Search strategy

We searched PubMed/MEDLINE, Scopus, Cochran, and Google Scholar from January 2004 till 16^th^ August 2024, in any language. The search terms were “Probiotics” and “Multiple sclerosis” Multiple sclerosis’. The syntax written based on the MeSH database and free text methods in PubMed database are (“multiple sclerosis”[MeSH Terms] OR (“multiple”[All Fields] AND “sclerosis”[All Fields]) OR “multiple sclerosis”[All Fields] OR “encephalomyelitis, autoimmune, experimental”[MeSH Terms] OR (“encephalomyelitis”[All Fields] AND “autoimmune”[All Fields] AND “experimental”[All Fields]) OR “experimental autoimmune encephalomyelitis”[All Fields] OR (“experimental”[All Fields] AND “autoimmune”[All Fields] AND “encephalomyelitis”[All Fields])) AND (“probiotic s”[All Fields] OR “probiotical”[All Fields] OR “probiotics”[MeSH Terms] OR “probiotics”[All Fields] OR “probiotic”[All Fields]). The duplicates were removed using EndNote X7 (Thomson Reuters, New York, NY, USA). The PRISMA guidelines were followed to perform this study (S1 File) [20].

### Ethical statement

This study was carried out with the Code of Ethics Committee (No. IR. KUMS. REC.1400.170) adopted by Kermanshah University of Medical Sciences and also project number 4000263.

### Inclusion/Exclusion criteria

Animal and human studies, including those on different probiotics, have been conducted. Suitable articles were selected on the basis of certain criteria. The excluded articles were systematic reviews, meta-analyses, or narrative reviews. All studies were reviewed separately by two authors, and a third expert re-reviewed and decided on inconsistencies. Studies were included if they met all of the following criteria:1) papers using single or multiple strains of probiotics with doses of 1 × 10^8^ - 3 × 10^11^ CFU and 1-26 weeks’ treatment duration, 2) papers that measured the effects of probiotics on MS patients, 3) assessment of inflammation in multiple sclerosis patients, 4) papers based on the correct method, and 5) papers that included animal groups without probiotic treatment or matched control human groups.

### Data extraction

After omission of some articles based on the criteria, the related data were extracted by two authors from an article about MS, probiotics, and the effect of probiotics, including 1) characteristics of the sample, 2) the probiotic strain used, 3) scales of effect measurement, 4) results of research, and 5) publication date. We classified data based on probiotic genus, dose of bacteria, period of treatment, examined factors, EAE Induction method, type of sample, and increase or reduction of inflammation.

### Risk of bias and/or quality assessment

The Critical Appraisal tools of Joanna Briggs Institute (JBI) were used to assess the quality (risk of bias) of each study [21]. Two reviewers independently completed the study, based on the checklist. Any inconsistencies were assessed by a third reviewer. The final quality of the studies was determined by summing the scores based on the checklist (S1 and S2 Tables). In addition, the SYRCLE’s risk of bias tool was used for animal studies [22] (S3 Table).

### Statistical analysis

A meta-analysis was performed Using Comprehensive Meta-Analysis software (version 2.2.064) to estimate the effect of probiotics on cytokine levels, immune cell percentages, and general outcomes (clinical score or EDSS). The studies were divided into animal and human study groups, and the analysis was performed separately. Because there was significant heterogeneity between studies, the random-effects model analysis was selected for all meta-analyses. The sample sizes of the probiotic treatment and control groups, their values (mean) for each index, and their standard deviations (SDs) were used to calculate the standard differences in means (Std diff in means). The outcomes are shown as mean ±  95% confidence interval (95% CIs). Q- and I-squared statistical tests were used to calculate heterogeneity between the reports. A Q-value greater than the number of studies minus one (with a p-value lower than 0.05) was considered to indicate the existence of heterogeneity. The amount of heterogeneity is shown by an I-squared number. Due to the small number of reports, the subgroup analysis was only applicable for animal studies reporting clinical scores, in which a subgroup analysis based on the method was performed. In addition, publication bias was assessed only for the clinical score data, as it is supported by a larger body of research and offers a more robust dataset compared to other outcomes, by creating funnel plots of standard error or precision by standard difference in mean as well as using Egger’s regression test. Duval and Tweedie’s trim and fill method was used to analysis the sensitivity of the publication bias. In all analyses, a p-value of less than 0.05 was accepted as significant.

## Results

### Literature search to identify case-controlled studies

**Fig 1:**
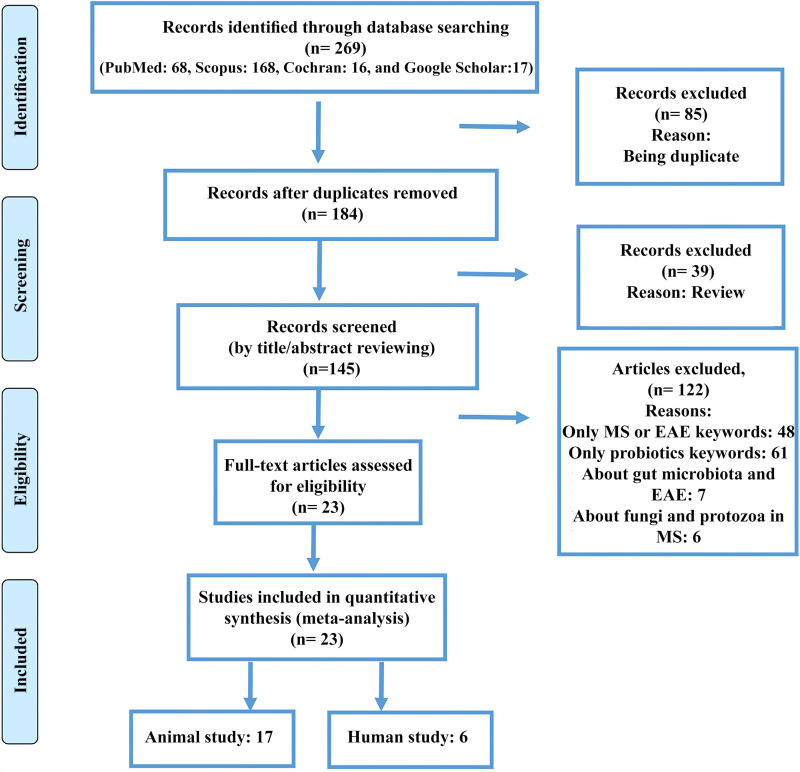
Systematic literature review flow diagram.

According to our search keywords, 269 articles were retrieved from the databases (PubMed: 68, Scopus: 168, Cochran: 16, and Google Scholar: 17), while 124 duplicated papers, narrative reviews, systematic reviews, and meta-analyses were omitted (Fig 1). The remaining 145 articles were screened and 48 articles were discussing only MS or EAE, 61 papers were only about probiotics and not their relationship with MS, seven articles were about gut microbiota and EAE, and six articles focused on the relationship between fungi and protozoa in MS, which was not our concern (S4 Table). Among the remaining 23 papers, 17 papers surveyed animal models, and six articles were human studies (Table 1).

**Table 1:** The characteristics of the studies.

Study Animal	Authors	Probiotics	NC^*^	NT¥	IFN-γ		IL-17		IL-10		TNF-α		Clinical score		EDSS		CD4 CD25		Method	Treg	
					Control	Test	Control	Test	Control	Test	Control	Test	Control	Test	Control	Test	Control	Test	ELISA	Control	Test
	Dargahi et al. (2020) [23]	*S. thermophiles*	3	3	62.2 ± 9.3	52.8 ± 8.4	N/A	N/A	14.49 ± 0.9	41.63 ± 5.09	17.85 ± 1.7	16.15 ± 1.02	N/A	N/A					ELISA		
	Digehsara.et. al (2020) [24]	*L. casei*	N/A	N/A	N/A	N/A	335.27 ± 32.19	145.59 ± 32.19	N/A	N/A	N/A	N/A	N/A	N/A					ELISA		
	He et al. (2019) [25]	*L. reuteri*	39	40	53 ± 10.82	26.84 ± 4.71	12.43 ± 2.66	5.20 ± 0.73	N/A	N/A	N/A	N/A	2.83 ± 0.04	2.02 ± 0.03					ELISA		
	Kobayashi et al. (2012) [26]	*L. casei*	13	14	772.2 ± 395.3	898.05 ± 832.9	614.25 ± 314.3	600.1 ± 285.7	419.04 ± 85.6	533.3 ± 71.4	N/A	N/A	1.79 ± 1.37	1.29 ± 1.59			1.53 ± 0.29	1.99 ± 0.29	ELISA, Flowcytometry		
	Lavasani et al. (2010) [27]	*Lactobacillus*	18	18	4153.85 ± 1200	415.38 ± 138.46	295.65 ± 38.25	193.13 ± 24.34	29 ± 18.3	164.88 ± 35.12	902.3 ± 171.4	126.3 ± 18.04	5.32 ± 0.52	1.39 ± 0.43					ELISA		
	Mangalam et al. (2017) [28]	*Prevotella, E. coli*	N/A	20	1036.08 ± 92.79	865.979 ± 46.39	587.62 ± 108.247	139.175 ± 30.92	46.39 ± 15.46	340.206 ± 46.39	46.3 ± 15.46	46.39 ± 30.92	2.77 ± 0.57	1.04 ± 0.44					ELISA		
	Rezende et al. (2013) [29]	*L. lactis*	10	10	13444.8 ± 3677.9	26838.7 ± 3612.9	3223.8 ± 778	3133.7 ± 1263.9	485.23 ± 147.9	880.5 ± 288.46	N/A	N/A	3.1 ± 0.25	1.48 ± 0.29					ELISA		
	Sadeghi et al. (2022) [30]	*B. coagulans*	7	7	421.1 ± 56.6	58.8 ± 43.02	332.3 ± 33.09	181.2 ± 10.07	N/A	N/A	N/A	N/A ± N/A	N/A	N/A					ELISA		
	Saisai et al. (2021) [31]	*L. acidipiscis,*	5	5	870.06 ± 62.9	484.6 ± 11.4	898.2 ± 63.1	371.9 ± 28.07	87.03 ± 8	277.1 ± 14	N/A	N/A	2.5 ± 0.27	0.28 ± 0.39					ELISA	6.7 ± 0.84	12.97 ± 0.34
		*E. coli*																			
	Salehipour et al. (2017) [32]	*L. plantarum,*	8	26	4619.05 ± 809.52	952.381 ± 809.51	7457 ± 1152.54	2237.29 ± 1152.54	50.4 ± 17.89	141.463 ± 19.51	N/A	N/A	2.56 ± 0.51	0.92 ± 0.4			6.9 ± 1	13.7 ± 2.7	ELISA, Flowcytometry	6.9 ± 1	13.7 ± 2.7
		*B. animalis*																			
	Samani et al. (2022) [33]	*E. durans,*	6	6	2.5 ± 0.07	2.7 ± 0.19	51.5 ± 2	39.75 ± 2.5	N/A	N/A	N/A	N/A	N/A	N/A					ELISA		
		*lacto-mix*																			
	Secher et al. (2017) [34]	*E. coli*	40	40	120000 ± 19000	44047.6 ± 10714.3	15471.7 ± 3773.6	9622.6 ± 1886.7	11.34 ± 1.62	22.68 ± 3.47	412.03 ± 46.3	342.59 ± 41.66	2.13 ± 0.15	0.88 ± 0.15					ELISA		
	Takata et al. (2011) [35]	*P. acidilactici*	17	18	7062 ± 1500	4750 ± 1187	3593 ± 532	1406 ± 250	400 ± 53	335 ± 46	N/A	N/A	3.11 ± 0.38	2.24 ± 0.41					ELISA		
	Abdurasulova et al. (2016) [36]	*E. faecium*	52	35													2.9 ± 0.92	1.32 ± 0.32	Flowcytometry		
	Calvo-Barreiro et al. [36]	*Bifidobacterium, Streptococcus, Lactobacillus*	8	24															Flowcytometry	6.02 ± 1.11	9.05 ± 2.14
	Kown et al. (2013) [36]	*Lactobacillus, Bifidobacterium,* *Streptococcus*	10	10	1 ± 0.1	0.28 ± 0.11	1 ± 0.1	1.09 ± 0.15	1 ± 0.1	3.9 ± 0.13	1 ± 0.1	0.2 ± 0.12	1.81 ± 0.55	2.52 ± 0.29							
	Ibrahim et al. (2023) [37]	*Bacillus*	10	10			151 ± 7.2	128.5 ± 11.2			72 ± 3.5	26.1 ± 3.2	4.32 ± 0.54	2.41 ± 0.27					ELISA		
	Montgomery et al. (2022) [38]	*Lactobacillus reuteri*	9	10			9.4 ± 0.94	21.9 ± 0.94											Flowcytometry		
**Study** **Human**	Kouchaki et al. (2017) [39]	*Lactobacillus, Bifidubacterium*	30	30											2.3 ± 0.1	2 ± 0.1			ELISA		
	Salami et al. (2019) [40]	*Lactobacillus, Bifidubacterium*	24	24	N/A	N/A	N/A	N/A	1.65 ± 0.15	2.88 ± 0.2	2.7 ± 0.26	2.82 ± 0.22	N/A	N/A	3 ± 0.19	2.33 ± 0.17	N/A	N/A	ELISA		
	Rahimlou et al. (2020) [41]	*Bacillus, Bifidobacterium, Lactobacillus, Streptococcus*	35	35	N/A	N/A	N/A	N/A	N/A	N/A	N/A	N/A	N/A	N/A	1.36 ± .0.19	1.23 ± 0.16	N/A	N/A	N/A		
	Rahimlou et al. (2022) [42]	probiotics capsules include *Bacillus, Bifidobacterium, Lactobacillus, Streptococcus strains*	33	32	2.88 ± 0.71	1.99 ± 0.25	2.50 ± 1.3	2.54 ± 1.4	N/A	N/A	0.516 ± 0.36	0.316 ± 0.27	N/A	N/A	1.39 ± 1.03	1.45 ± 0.9	N/A	N/A	ELISA	N/A	N/A
	Chakamian et al. (2023) [43]	*Lactobacillus strains, L paracasei* DSM 13434 and *L. plantarum* DSM 15312	9	21	6.92 ± 11.28	44.86 ± 13.9	445.5 ± 64.18	61.23 ± 28.63	N/A	N/A	N/A	N/A	N/A	N/A	N/A	N/A	N/A	N/A	ELISA	N/A	N/A
	Hosseini et al. (2018) [44]	*B. subtilis, B. bifidum, B. breve, B. infantis, B. longum, L. acidophilus, L. delbrueckii, L. casei, L. plantarum, L. rhamnosus, L. helveticus, L. salivarius, L. lactis, S. thermophilus*	32	33	2.883 ± 0.71	1.998 ± 0.25	2.504 ± 1.3	2.546 ± 1.4	1.65 ± 0.15	2.88 ± 0.2	0.516 ± 0.36	0.316 ± 0.27	N/A		1.72 ± 0.74	1.68 ± 0.71					

*NC: Number of control.

¥ NT: Number of treatments.

### Animal studies

The indices reported in animal studies were the production of IFN-γ, IL-17, IL-10, and TNF-α (obtained by either enzyme-linked immunosorbent assay or ELISA, flow cytometry, and reverse transcription-polymerase chain reaction (RT-PCR)), the percentage of regulatory T cells (Treg) and CD4 CD25 cells (by flow cytometry), and the clinical scores.

### IFN-gamma production

**Fig 2:**
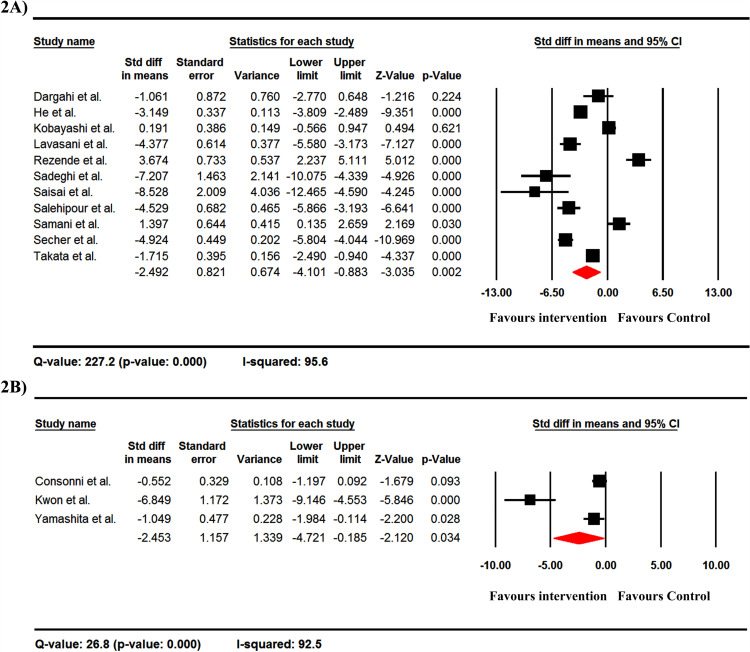
The forest plots of the effect of probiotics on the level of IFN-γ by ELISA (A) and RT-PCR (B) methods. It should be noted that these data are from EAE studies. Overall, probiotics decreased IFN-γ production with Std diff in means between probiotics and control groups was -2.492 with ELISA test (A) and -2.453 with RT-PCR test (B).

Overall, probiotics decreased IFN-γ production with Std diff in means between probiotics and control groups was -2.492 with ELISA tests (Fig 2A) and -2.453 with RT-PCR test (Fig 2B). It is noteworthy that only one study reported IFN-γ using the flow cytometry method; hence, the analysis of that study was not applicable. Significant heterogeneity was observed between studies (as shown by the Q-value and I-squared value in Fig 2A and 2B).

### IL-17 production

**Fig 3:**
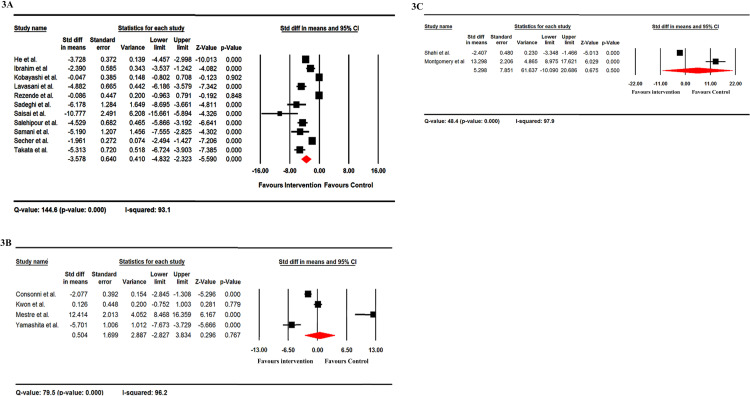
The forest plots of the effect of probiotics on the level of IL-17 by ELISA (A), RT-PCR (B) and Flow cytometry (C) methods. It should be noted that these data are from EAE studies. The overall level of IL-17 decreased after probiotic addition in ELISA test (overall Std diff in means = -3.578) (A), but increased following probiotic administration in RT-PCR (overall Std dif in means = 0.504) (B), and flow cytometry (overall Std dif in means = 5.298) (C).

The overall level of IL-17 decreased after probiotic addition in ELISA tests (overall Std diff in means = -3.578) (Fig 3A), but increased following probiotic administration in RT-PCR (overall Std dif in means = 0.504) (Fig 3B), and flow cytometry (overall Std dif in means = 5.298) (Fig 3C). There was significant heterogeneity between studies regarding IL-17 production, indicated by the Q-value and I-squared value (Fig 3A, 3B, and 3C).

### IL-10 production

**Fig 4:**
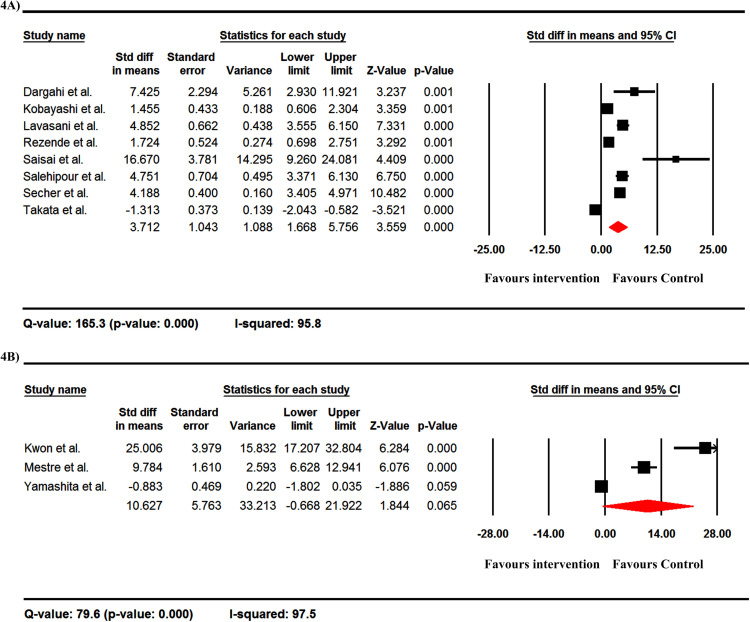
The forest plots of the effect of probiotics on the level of IL-10 by ELISA (A) and RT-PCR (B) methods. These data are from EAE studies. The level of IL-10 increased after the consumption of probiotics and the overall standard difference in means was 3.712 (A) and 10.627 (B) in ELISA and RT-PCR studies, respectively.

In both ELISA- and RT-PCR-based studies, the level of IL-10 was increased after taking probiotics, and the overall Std dif in means was 3.712 (Fig 4A) and 10.627 (Fig 4B) in ELISA and RT-PCR studies, respectively. None of the studies used flow cytometry to assess the IL-10 levels. In addition, there was significant heterogeneity between reports of IL-10 levels (Fig 4A and 4B).

### TNF-α production

**Fig 5:**
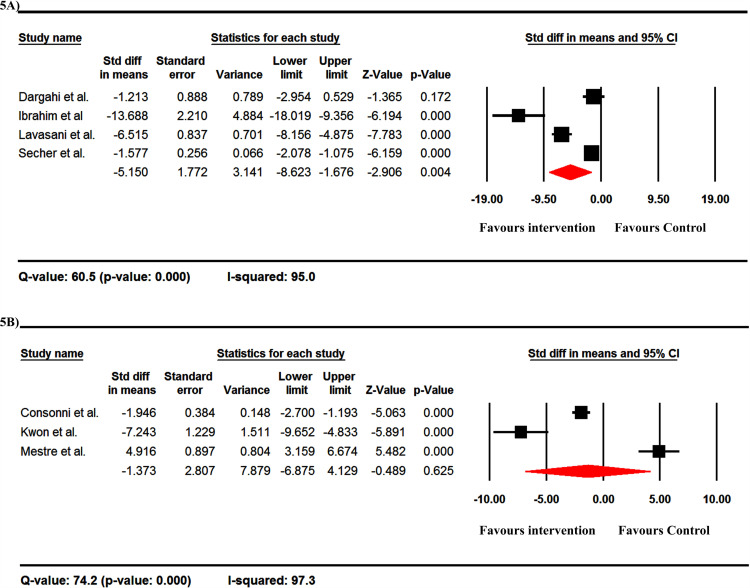
The forest plots of the effect of probiotics on the level of TNF-α by ELISA (A) and RT-PCR (B) methods. These data are from EAE studies. The probiotics reduced the TNF-α level with an overall standard difference in means between groups of -3.060 in ELISA test (A) and -1.373 in RT-PCR test (B).

Overall, the probiotics reduced the TNF-α level with an overall Std diff in means between groups of -3.060 in ELISA tests (Fig 5A) and -1.373 in RT-PCR test (Fig 5B). Flow cytometry was not used to assess TNF-α levels. Significant heterogeneity was observed between the studies (Fig 5A and 5B).

### T-reg and CD4/CD25 cell percentage

**Fig 6:**
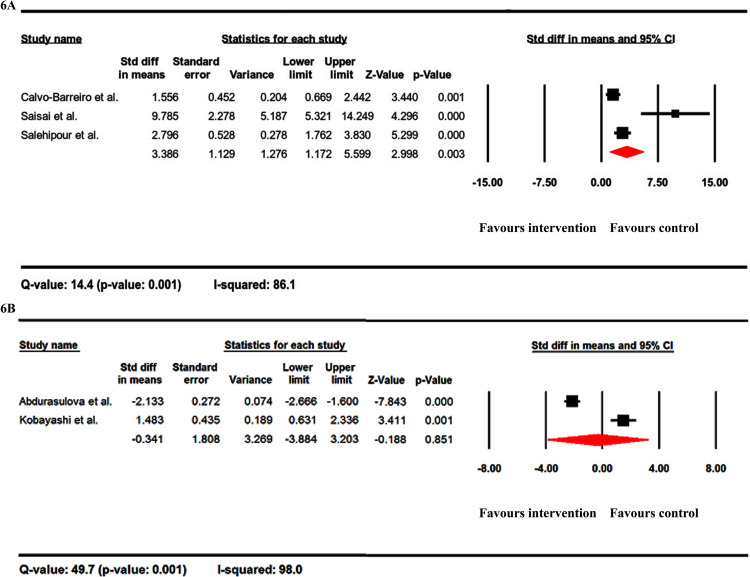
The forest plots of the effect of probiotics on the level of T-reg (A) and CD4 CD25 (B). These data are from EAE studies. Based on the results, the standard difference in means between probiotics-takers and control groups was 3.386 for Treg cells (A) and -0.290 for CD4 CD25 cells (B).

The number of Treg and CD4/CD25 cells was reported by flow cytometry in three and two studies, respectively. Probiotics increased the percentage of Treg cells but decreased the percentage of CD4/CD25 cells. The overall Std difference in means between probiotics-takers and control groups was 3.386 for Treg cells (Fig 6A) and -0.290 for CD4 CD25 cells (Fig 6B).

### Clinical scores

**Fig 7:**
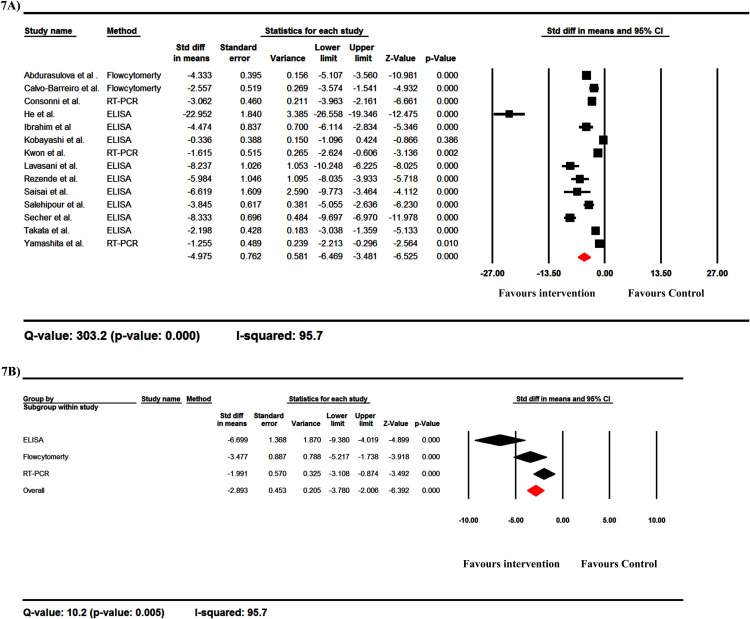
The forest plots of the effect of probiotics on clinical score. These data are from EAE studies. The clinical score was lower in the probiotic-received group than in the control group, and the overall standard difference in the means between the two groups was -4.975 (95%CIs: -6.469, -3.481) (A). A subgroup analysis of clinical scores based on the method used showed that in all methods, the clinical scores were lower in the probiotic-received group than in the control group, with higher differences detected by ELISA (B).

The clinical scores were reported in 14 studies. Meta-analysis of this study showed that the clinical score was lower in the probiotic-received group than in the control group, and the overall standard deviation in the means between the two groups was -4.975 (95%CIs: -6.469, -3.481) (Fig 7A). A subgroup analysis of clinical scores based on the method used showed that in all methods, the clinical scores were lower in the probiotic-received group than in the control group, with higher differences detected by ELISA (Fig 7B).

### Publication bias

**Fig 8:**
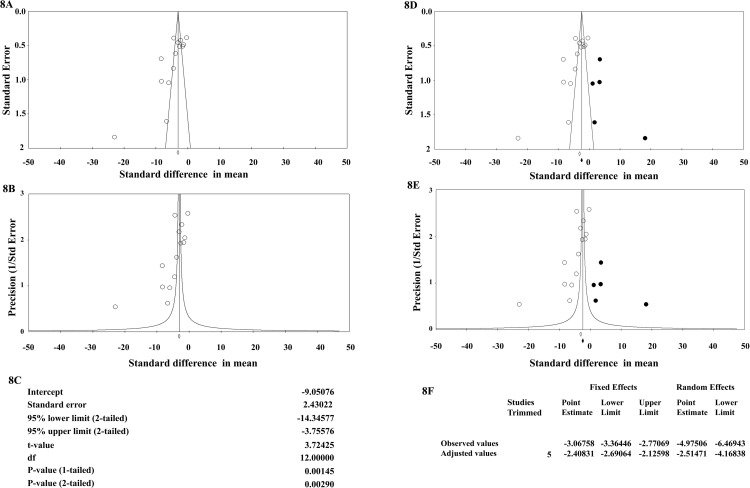
The publication bias assessment and sensitivity analysis. The assessment of publication bias was done by drawing the funnel plots of standard error (A) or precision (B) by standard difference in mean as well as using Egger’s regression test (C). The asymmetric distribution of the studies (open circles) over the combined effect size can be observed. The sensitivity assay of publication bias was done by Duval and Tweedie’s trim and fill method which suggests that five studies are missing. These five studies are shown by solid black circles in both funnel plots of standard error (D) or precision (E). The observed and adjusted effect sizes as well as other statistical parameters of the trim and fill method are also shown (F).

Considering clinical score data, publication bias was assessed by drawing funnel plots and applying the Egger’s test. The both funnel plots of standard error or precision by standard difference showed asymmetric distribution of studies suggesting a potential publication bias. Also, the Egger’s test showed significant publication bias since the 1-tailed p-value (recommended by Comprehensive Meta-Analysis software) is 0.00145, and the 2-tailed p-value is 0.00290 (Fig 8).

The Duval and Tweedie’s trim and fill method trims the asymmetric studies from the one side to locate the unbiased effect, and then fills the plot by re-inserting the trimmed studies on that side as well as their imputed counterparts to the other side of the mean effect. In the present study we chose “the right of mean” as where the missing studies should be looked for. Using these parameters, the method suggests that 5 studies are missing. Under the fixed effect model the point estimate and 95% confidence interval for the combined studies is -3.06758 (-3.36446, -2.77069). Using Trim and Fill the imputed (adjusted) point estimate is -2.40831 (-2.69064, -2.12598). Under the random effects model the point estimate and 95% confidence interval for the combined studies is -4.97506 (-6.46943, -3.48067). Using Trim and Fill the imputed point estimate is -2.51471 (-4.16838, -0.86105). The imputed studies are depicted in the funnel plots (Fig 8). These results show that studies that show the reducing effect of probiotics on MS have a higher probability of being published.

### Human studies

**Fig 9:**
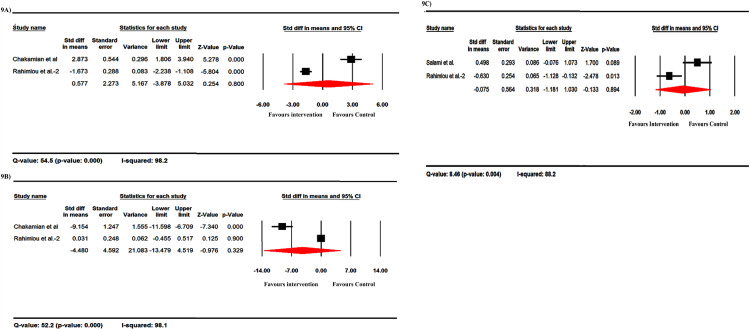
The forest plots of the effect of probiotics in human studies on the level of IFN-γ (A), IL-17 (B), and TNF-α (C). IFN-γ was increased following probiotic administration as the Std diff in means equal to 0.577 (A). The IL-17 and TNF-α were decreased after probiotic administration with the Std diff in means equal to -4.480 and -0.075, respectively (B and C).

**Fig 10:**
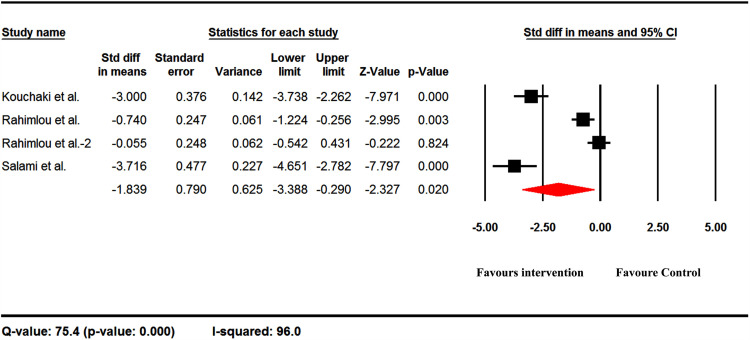
Probiotic consumption in people with MS had favorable effects on EDSS. The overall EDSS was lower in probiotics-received subjects than in controls, with the Std diff in means equal to -1.839.

There are few reports on humans for some of the studied indices, so that the analysis was only possible for IFN-γ, IL-17, and TNF-α levels (by ELISA) and EDSS. IFN-γ was increased following probiotic administration as the Std diff in means equal to 0.577 (Fig 9A). The IL-17 and TNF-α were decreased after probiotic administration with the Std diff in means equal to -4.480 and -0.075, respectively (Fig 9B and 9C). The overall EDSS was lower in probiotics-received subjects than in controls, with the Std diff in means equal to -1.839 (Fig 10).

## Discussion

It seems that the innate and adaptive arms of the immune system (innate and adaptive) play an important role in the pathogenesis of MS [45]. Microglia and macrophages are professional phagocytic cells found in central nervous system (CNS) lesions in both MS and EAE models [46, 47]. These cells are mainly responsible for the damage and removal of myelin in MS lesions [46, 48]. In contrast, a significant proportion of CD4^ +^ and CD8^ +^ lymphocytes isolated from MS lesions showed an antigen-specific T cell response contributing to the disease process [49]. Th1 and Th17 cells promote inflammation of the CNS, while Th 2 and T-reg cells inhibit it [50]. In addition, the balance between pro-inflammatory Th1/Th17 cells and anti-inflammatory CD4^ + ^FoxP3^ +^ regulatory T cells shifts toward a pro-inflammatory response [51]. Therefore, it is possible to affect this balance, return to an anti-inflammatory response, and finally suppress the disease using potential therapeutic factors.

MS and EAE are associated with the overexpression of cytokines such as IL-12, IFN-γ, IL-6, IL-1β, IL-21, and IL-23, which are partially effective in promoting the differentiation of Th1 and Th17 cells [52].

Probiotics regulate both innate and adaptive host immune response mechanisms [53, 54]. The anti-inflammatory role of probiotics is mediated by increased levels of IL-4, transforming growth factor beta (TGF-β), IL-10 cytokines, and T-reg cells, leading to improved EAE conditions. In addition, consumption of probiotics reduces the severity of EAE by reducing Th1/Th17 levels [32]. In addition to enhancing the host immune system, probiotics have important beneficial effects in the prevention and/or management of immune-related diseases, including inflammatory bowel disease, irritable bowel syndrome, diarrhea, bacterial infections, and certain types of cancer.

The development of new therapeutic strategies to improve MS pathology requires an understanding of the mechanisms of cytokine-mediated CNS damage. IFN-γ, as an important cytokine product of the Th1-type immune response, plays an important role in immune-mediated demyelination diseases such as MS because its level increases strongly during the activity of this disease [55, 56]. In this meta-analysis, it was shown that the consumption of probiotics in patients with MS has a decreasing effect on IFN-γ levels. In other words, probiotics lead to significant clinical improvement in patients with MS.

A decrease in anti-inflammatory IL-10 levels is associated with increased MS severity [57]. On the other hand, increased induction of IL-10 by *Escherichia coli* strain Nissle 1917-treated mice may be a preventive mechanism of inflammation and autoimmunity [58, 59]. Accordingly, in the eight analyzed studies, the results indicated that probiotics increased IL-10 levels.

MS can cause mortality, inflammation, dyslipidemia, high blood pressure, and insulin resistance. Based on our analysis of human studies, probiotic supplementation in patients with MS decreased TNF-α gene expression. In addition, probiotic supplementation significantly reduced EDSS scores (SD =  -1.839) in people with MS.

Probiotics can stimulate beneficial Treg cells to maintain immune balance [60]. For example, *L. plantarum* WCFS1 associated with CD103^ +^ DC shows its activity in a way that by penetrating the intestine, it increases Th2 cytokines and finally stimulates the production of Treg cells [61]. The findings shown in Fig 6 indicate that consumption of probiotics can stimulate Treg cells.

Finally, IL-17, an inflammatory marker in MS, has been shown to decrease with the administration of probiotics (mono-strain in 8 studies and a mixture of probiotics in 4 animal studies). However, it has only been examined in one human study, which does not demonstrate a significant effect on its level [27–38,41,45]. Conversely, IL-10, an inhibitory cytokine that is reduced in MS patients, increased with the administration of probiotics in 10 animal studies (monostrain in 6 and mixture in 4 studies) and 2 human studies, which may help improve MS symptoms [26,29–32,34,35,37,38,41,43,45]. Additionally, IFN-γ decreased in 10 animal studies (6 with probiotic alone and 4 with probiotics in combination) and one human study [26,28,30,31,33–35,37,38,41,45], while it increased in 3 animal studies [29,32,36]. Regarding TNF-α, no increase was observed in the animal model with the administration of probiotics, but a decrease in this inflammatory cytokine was reported in 4 studies [26,30,37,41]. In human studies, it was increased in one case and decreased in another [43,45].

## Conclusion

Twenty-seven animal and seven human studies systematically demonstrated the effects of probiotic consumption on MS. These findings indicate that probiotic consumption may have potentially beneficial effects in reducing the incidence and severity of MS and in delaying disease progression. Since probiotic therapy may be considered a promising opportunity for the treatment of MS, physicians and nutritionists should consider approved probiotic supplements to manage health concerns in MS.

## Supporting information

S1 File The PRISMA guidelines were followed to conduct this study. (DOCX)

S1 Table The JBI critical appraisal checklist for qualitative research. (DOCX)

S2 Table JBI critical appraisal checklist for randomized controlled trials. (DOCX)

S3 Table SYRCLE’s risk of bias checklist for animal studies .
(DOCX)

S4 Table Inclusion/Exclusion criteria 186 studies were included in the present study. (DOCX)
