# Supplementary material for: The potential effectiveness of probiotics in reducing multiple sclerosis progression in preclinical and clinical studies: A worldwide systematic review and meta-analysis
Source: PLoS One. 2025 Apr 24;20(4):e0319755. doi: 10.1371/journal.pone.0319755 (PMC12021188; doi:10.1371/journal.pone.0319755)
Supplement: S1 Table — (DOCX) [file pone.0319755.s002.docx]

| S1 Table. JBI critical appraisal checklist for qualitative research | | | | | | | | | | | |
| --- | --- | --- | --- | --- | --- | --- | --- | --- | --- | --- | --- |
| **Author(s)** | Q1 | Q2 | Q3 | Q4 | Q5 | Q6 | Q7 | Q8 | Q9 | Q10 | Total |
| Dargahi et al. | Yes | Yes | Yes | Yes | Yes | Unclear | No | No | Yes | Yes | 7 |
| Digehsara.et. al | Yes | Yes | Yes | Yes | Yes | Unclear | No | Yes | Yes | Yes | 8 |
| He et al. (2019) | Yes | Yes | Yes | Yes | Yes | No | No | Yes | Yes | Yes | 8 |
| Kobayashi et al. (2012) | Yes | Yes | Yes | Yes | Yes | No | No | Yes | Unclear | Yes | 7 |
| Lavasani et al. (2010) | Yes | Yes | Yes | Yes | Yes | Unclear | No | Yes | Yes | Yes | 8 |
| Mangalam et al. (2017) | Yes | Yes | Yes | Yes | Yes | No | No | Yes | Yes | Yes | 8 |
| Rezende et al. (2013) | Yes | Yes | Yes | Yes | Yes | Unclear | No | Unclear | Yes | Yes | 7 |
| Sadeghi et al. (2022) | Yes | Yes | Yes | Yes | Yes | No | No | Yes | Yes | Yes | 8 |
| Saisai et al. (2021) | Yes | Yes | Yes | Yes | Yes | No | No | Yes | Yes | Yes | 8 |
| Salehipour et al. (2017) | Yes | Yes | Yes | Yes | Yes | No | No | Yes | Yes | Yes | 8 |
| Samani et al. (2022) | Yes | Yes | Yes | Yes | Yes | Unclear | Unclear | Yes | Yes | Yes | 8 |
| Secher et al. (2017) | Yes | Yes | Yes | Yes | Yes | No | No | Yes | Yes | Yes | 8 |
| Takata et al. (2011) | Yes | Yes | Yes | Yes | Yes | yes | No | Yes | Yes | Yes | 9 |
| Kouchaki et al. (2017) | Yes | Yes | Yes | Yes | Yes | Unclear | Unclear | Yes | Yes | Yes | 8 |
| Salami et al. (2019) | Yes | Yes | Yes | Yes | Yes | No | No | Yes | Yes | Yes | 8 |
| Rahimlou et al. (2020) | Yes | Yes | Yes | Yes | Yes | Unclear | No | No | Yes | Yes | 7 |
| Hosseini et al. (2018) | Yes | Yes | Yes | Yes | Yes | No | No | Yes | Yes | Yes | 8 |
| Chakamian et al. | Yes | Yes | Yes | Yes | Yes | Yes | Unclear | Yes | Yes | Yes | 9 |
| Rahimlou et al. | Yes | Yes | Yes | Yes | Yes | No | No | Yes | Yes | Yes | 8 |
