# Supplementary material for: The potential effectiveness of probiotics in reducing multiple sclerosis progression in preclinical and clinical studies: A worldwide systematic review and meta-analysis
Source: PLoS One. 2025 Apr 24;20(4):e0319755. doi: 10.1371/journal.pone.0319755 (PMC12021188; doi:10.1371/journal.pone.0319755)
Supplement: S2 Table — (DOCX) [file pone.0319755.s003.docx]

| S2 Table. JBI Critical Appraisal Checklist for Randomized Controlled Trials | | | | | | | | | | | | | | |
| --- | --- | --- | --- | --- | --- | --- | --- | --- | --- | --- | --- | --- | --- | --- |
| **Author(s)** | Q1 | Q2 | Q3 | Q4 | Q5 | Q6 | Q7 | Q8 | Q9 | Q10 | Q11 | Q12 | Q13 | Total |
| Kouchaki et al. (2017) | Yes | Yes | Yes | Yes | Unclear | Yes | Yes | Yes | Yes | Yes | Yes | Yes | Yes | 12 |
| Salami et al. (2019) | Yes | Yes | Yes | Yes | Yes | Yes | Yes | Yes | Yes | Yes | Yes | Yes | Yes | 13 |
| Rahimlou et al. (2020) | Yes | Yes | Yes | Yes | Yes | Yes | Yes | Unclear | Yes | Yes | Yes | Yes | Yes | 12 |
| Hosseini et al. (2018) | Yes | Yes | Yes | Yes | Yes | Yes | Yes | Yes | Yes | Yes | Yes | Yes | Yes | 13 |
| Chakamian et al. | Yes | Yes | Yes | Yes | Unclear | Unclear | Yes | Yes | Yes | Yes | Yes | Yes | Yes | 11 |
| Rahimlou et al. | Yes | Yes | Yes | Yes | Yes | Yes | Yes | Yes | Yes | Yes | Yes | Yes | Yes | 13 |
