# Supplementary material for: The potential effectiveness of probiotics in reducing multiple sclerosis progression in preclinical and clinical studies: A worldwide systematic review and meta-analysis
Source: PLoS One. 2025 Apr 24;20(4):e0319755. doi: 10.1371/journal.pone.0319755 (PMC12021188; doi:10.1371/journal.pone.0319755)
Supplement: S3 Table — . (DOCX) [file pone.0319755.s004.docx]

| S3 Table. SYRCLE's risk of bias checklist for animal studies | | | | | | | | | | | |
| --- | --- | --- | --- | --- | --- | --- | --- | --- | --- | --- | --- |
| **Author(s)** | Q1 | Q2 | Q3 | Q4 | Q5 | Q6 | Q7 | Q8 | Q9 | Q10 | Total |
| Dargahi et al. (2020) | Yes | Yes | Yes | Unclear | No | Yes | Yes | No | Yes | Yes | 7 |
| Digehsara.et. al (2020) | Yes | Yes | Yes | No | No | Yes | Yes | No | Yes | Yes | 7 |
| He et al. (2019) | Yes | Yes | Yes | Yes | No | Yes | Yes | No | Yes | Yes | 8 |
| Kobayashi et al. (2012) | Yes | Yes | Yes | Unclear | Yes | Yes | Yes | No | Yes | Yes | 8 |
| Lavasani et al. (2010) | Yes | Yes | Yes | Unclear | No | Yes | Yes | No | Yes | Yes | 7 |
| Mangalam et al. (2017) | Yes | Yes | Yes | Unclear | No | Yes | Yes | No | No | Yes | 6 |
| Rezende et al. (2013) | Yes | Yes | Yes | Unclear | Yes | Yes | Yes | Unclear | Yes | Yes | 8 |
| Sadeghi et al. (2022) | Yes | Yes | Yes | Yes | No | Yes | Yes | No | Yes | Yes | 8 |
| Saisai et al. (2021) | Yes | Yes | Yes | No | No | Yes | Yes | No | Yes | Yes | 7 |
| Salehipour et al. (2017) | Yes | Yes | Yes | No | No | Yes | Yes | No | Yes | Yes | 7 |
| Samani et al. (2022) | Yes | Yes | Yes | Unclear | No | Yes | Yes | No | Yes | Yes | 7 |
| Secher et al. (2017) | Yes | Yes | Yes | Yes | No | Yes | Yes | No | Yes | Yes | 8 |
| Takata et al. (2011) | Yes | Yes | Yes | Yes | Unclear | Yes | No | No | Yes | Yes | 7 |
| Abdurasulova et al. (2016) | Yes | Yes | yes | Unclear | No | Yes | Yes | No | Yes | Yes | 7 |
| Calvo-Barreiro et al. | Yes | Yes | Yes | Unclear | Yes | Yes | Unclear | No | Yes | Yes | 7 |
| Ibrahim et al. (2023) | Yes | Yes | Yes | Yes | Unclear | Yes | No | No | Yes | Yes | 7 |
| Montgomery et al. (2022) | Yes | Yes | Yes | Unclear | Yes | Yes | Unclear | Yes | Yes | Yes | 8 |
