# Supplementary material for: The potential effectiveness of probiotics in reducing multiple sclerosis progression in preclinical and clinical studies: A worldwide systematic review and meta-analysis
Source: PLoS One. 2025 Apr 24;20(4):e0319755. doi: 10.1371/journal.pone.0319755 (PMC12021188; doi:10.1371/journal.pone.0319755)
Supplement: S4 Table — (DOCX) [file pone.0319755.s005.docx]

S4 Table. Inclusion/Exclusion criteria 186 studies were included in the present study.

| Authors | **Reason** | **Number** |
| --- | --- | --- |
| **Stoiloudis et al.(2022)** | **Review** | **1** |
| **Baecher-Allan et al.(2018)** | **Review** | **2** |
| **Dynka et al.(2022)** | **Review** | **3** |
| **Christovich et al.(2022)** | **Review** | **4** |
| **Yang et al.(2022)** | **Review** | **5** |
| **Nourbakhsh et al.(2019)** | **Review** | **6** |
| **Sorboni et al.(2022)** | **Review** | **7** |
| **Cryan et al.(2020)** | **Review** | **8** |
| **Dunalska et al.(2023)** | **Review** | **9** |
| **Rutsch et al.(2020)** | **Review** | **10** |
| **Shimokawa C.(2025)** | **Review** | **11** |
| **Galland. (2014)** | **Review** | **12** |
| **Preiningerova et al.(2022)** | **Review** | **13** |
| **Belvoncikova et al.(2022)** | **Review** | **14** |
| **Ordonez-Rodriguez et al.(2023)** | **Review** | **15** |
| **Riccio et al.(2015)** | **Review** | **16** |
| **Miyauchi et al.(2022)** | **Review** | **17** |
| **Yamamoto et al.(2020)** | **Review** | **18** |
| **Correale et al.(2022)** | **Review** | **19** |
| **Parodi et al.(2021)** | **Review** | **20** |
| **Kujawa et al.(2023)** | **Review** | **21** |
| **Mirzaei et al.(2021)** | **Review** | **22** |
| **Fettig et al.(2024)** | **Review** | **23** |
| **Mehrabani et al.(2023)** | **Review** | **24** |
| **Jette et al.(2024)** | **Review** | **25** |
| **Wasko et al.(2020)** | **Review** | **26** |
| **Amini et al.(2020)** | **Review** | **27** |
| **Mowry et al.(2017)** | **Review** | **28** |
| **Hoogen et al.(2017)** | **Review** | **29** |
| [**Vaarala**](https://openurl.ebsco.com/results?sid=ebsco:ocu:record&bquery=AU+Vaarala,%20O.&link_origin=scholar.google.com)  **et al. (2003)** | **Review** | **30** |
| **Vitaliti et al.(2014)** | **Review** | **31** |
| **Tsai et al.(2012)** | **Review** | **32** |
| **Ozdemir et al.(2013)** | **Review** | **33** |
| **Dargahia et al.(2019)** | **Review** | **34** |
| **Li et al.(2020)** | **Review** | **35** |
| **Cheung et al.(2019)** | **Review** | **36** |
| **Morshedi et al.(2019)** | **Review** | **37** |
| **Plemel et al.(2015)** | **Review** | **38** |
| **Vlasova et al.(2016)** | **Review** | **39** |
| **Reason: Review** | | **Total :39** |
| Authors | **Reason** | **Number** |
| **Choi et al.(2016)** | **Only MS or EAE keywords** | **1** |
| **Mossakowski et al.(2015)** | **Only MS or EAE keywords** | **2** |
| **Dousset et al(2006)** | **Only MS or EAE keywords** | **3** |
| **Miyake et al.(2015)** | **Only MS or EAE keywords** | **4** |
| **Lomakin et al.(2016)** | **Only MS or EAE keywords** | **5** |
| **Offner et al.(2011)** | **Only MS or EAE keywords** | **6** |
| **Bar-Or et al.(2023)** | **Only MS or EAE keywords** | **7** |
| **Nicholson et al.(2023)** | **Only MS or EAE keywords** | **8** |
| **Monif et al.(2024)** | **Only MS or EAE keywords** | **9** |
| **Zurmati et al.(2023)** | **Only MS or EAE keywords** | **10** |
| **Feinstein et al.(2023)** | **Only MS or EAE keywords** | **11** |
| **Thomas et al.(2015)** | **Only MS or EAE keywords** | **12** |
| **Maghbooli et al.(2024)** | **Only MS or EAE keywords** | **13** |
| **Khodaie et al.(2023)** | **Only MS or EAE keywords** | **14** |
| **Putzki et al.(2009)** | **Only MS or EAE keywords** | **15** |
| **Prosperini et al.(2020)** | **Only MS or EAE keywords** | **16** |
| **Maurer et al.(2016)** | **Only MS or EAE keywords** | **17** |
| **Silva et al.(2022)** | **Only MS or EAE keywords** | **18** |
| **Essen et al.(2019)** | **Only MS or EAE keywords** | **19** |
| **Zhao et al.(2021)** | **Only MS or EAE keywords** | **20** |
| **Serada et al.(2008)** | **Only MS or EAE keywords** | **21** |
| **Yura et al.(2001)** | **Only MS or EAE keywords** | **22** |
| **Ahn et al.(2018)** | **Only MS or EAE keywords** | **23** |
| **Berer et al.(2017)** | **Only MS or EAE keywords** | **24** |
| **Matsushita et al.(2010)** | **Only MS or EAE keywords** | **25** |
| **Sovobe et al.(2007)** | **Only MS or EAE keywords** | **26** |
| **Mony et al.(2014)** | **Only MS or EAE keywords** | **27** |
| **Zhang et al.(2004)** | **Only MS or EAE keywords** | **28** |
| **Blazevski et al.(2013)** | **Only MS or EAE keywords** | **29** |
| **Skundric et al.(2005)** | **Only MS or EAE keywords** | **30** |
| **Burkhart et al.(1999)** | **Only MS or EAE keywords** | **31** |
| **Visser et al.(2005)** | **Only MS or EAE keywords** | **32** |
| **Genchi et al.(2022)** | **Only MS or EAE keywords** | **33** |
| **Reparaz et al.(2019)** | **Only MS or EAE keywords** | **34** |
| **Maassen et al.(2003)** | **Only MS or EAE keywords** | **35** |
| **Shahi et al.(2019)** | **Only MS or EAE keywords** | **36** |
| **Dousset et al.(2006)** | **Only MS or EAE keywords** | **37** |
| **Hossain et al.(2018)** | **Only MS or EAE keywords** | **38** |
| **Nazliel et al.(2002)** | **Only MS or EAE keywords** | **39** |
| **Calabrese et al.(2002)** | **Only MS or EAE keywords** | **40** |
| **Bhargava et al.(2017)** | **Only MS or EAE keywords** | **41** |
| **Zangeneh et al.(2021)** | **Only MS or EAE keywords** | **42** |
| **Hugos et al.(2024)** | **Only MS or EAE keywords** | **43** |
| **Col et al.(2023)** | **Only MS or EAE keywords** | **44** |
| **Minen et al.(2020)** | **Only MS or EAE keywords** | **45** |
| **Bowen et al.(2024)** | **Only MS or EAE keywords** | **46** |
| **Nezhad et al.(2024)** | **Only MS or EAE keywords** | **47** |
| **Hynes et al.(2025)** | **Only MS or EAE keywords** | **48** |
| **Reason: Only MS or EAE keywords** | | **Total: 48** |
| Authors | **Reason** | **Number** |
| **Baiao et al.(2023)** | **Only probiotics keywords** | **1** |
| **Boehme et al.(2023)** | **Only probiotics keywords** | **2** |
| **Hiraku et al.(2023)** | **Only probiotics keywords** | **3** |
| **Gedam et al.(2022)** | **Only probiotics keywords** | **4** |
| **Hanson et al.(2023)** | **Only probiotics keywords** | **5** |
| **Djaldetti et al.(2017)** | **Only probiotics keywords** | **6** |
| **Mariman et al.(2014)** | **Only probiotics keywords** | **7** |
| **Toscano et al.(2017)** | **Only probiotics keywords** | **8** |
| **Takahashi et al.(2006)** | **Only probiotics keywords** | **9** |
| **Cox et al.(2010)** | **Only probiotics keywords** | **10** |
| **Singh et al.(2018)** | **Only probiotics keywords** | **11** |
| **Yumi et al.(2009)** | **Only probiotics keywords** | **12** |
| **Meng et al.(2017)** | **Only probiotics keywords** | **13** |
| **Ekmekciu et al.(2017)** | **Only probiotics keywords** | **14** |
| **Gomes-Santos et al.(2017)** | **Only probiotics keywords** | **15** |
| **De Wolfe et al.(2018)** | **Only probiotics keywords** | **16** |
| **Smelt et al.(2012)** | **Only probiotics keywords** | **17** |
| **Ochoa –Reperaz et al.(2010)** | **Only probiotics keywords** | **18** |
| **Foligne et al.(2007)** | **Only probiotics keywords** | **19** |
| **Gackowsa et al.(2006)** | **Only probiotics keywords** | **20** |
| **Niers et al.(2005)** | **Only probiotics keywords** | **21** |
| **Castellazzi et al.(2007)** | **Only probiotics keywords** | **22** |
| **Srutkova et al.(20110** | **Only probiotics keywords** | **23** |
| **Desbonnet et al.(2008)** | **Only probiotics keywords** | **24** |
| **Mansour et al.(2014)** | **Only probiotics keywords** | **25** |
| **Ogita et al.(2015)** | **Only probiotics keywords** | **26** |
| **Ispirli et al.(2015)** | **Only probiotics keywords** | **27** |
| **Jensen et al.(2015)** | **Only probiotics keywords** | **28** |
| **Hlivac et al.(2005)** | **Only probiotics keywords** | **29** |
| **Siepert et al.(2014)** | **Only probiotics keywords** | **30** |
| **Tarasova et al.(2010)** | **Only probiotics keywords** | **31** |
| **Scharek et al.(2005)** | **Only probiotics keywords** | **32** |
| **Cohen–Poradosu et al.(2011)** | **Only probiotics keywords** | **33** |
| **Spaiser et al.(20150** | **Only probiotics keywords** | **34** |
| **Xia et al.(2018)** | **Only probiotics keywords** | **35** |
| **Li et al.(2011)** | **Only probiotics keywords** | **36** |
| **Nova et al.(2016)** | **Only probiotics keywords** | **37** |
| **Zarrati et al.(2013)** | **Only probiotics keywords** | **38** |
| **Tankou et al.(2018)** | **Only probiotics keywords** | **39** |
| **Consonni et al.(2018)** | **Only probiotics keywords** | **40** |
| **Troy et al.(2011)** | **Only probiotics keywords** | **41** |
| **Khalifa et al.(2023)** | **Only probiotics keywords** | **42** |
| **Yamashita et al.(2018)** | **Only probiotics keywords** | **43** |
| **Tankou et al.(2018)** | **Only probiotics keywords** | **44** |
| **Goudarzvand et al.(2016)** | **Only probiotics keywords** | **45** |
| **Kasarello et al.(2015)** | **Only probiotics keywords** | **46** |
| **Tamtaji et al.(2017)** | **Only probiotics keywords** | **47** |
| [**Sajedi**](https://onlinelibrary.wiley.com/authored-by/Sajedi/Donya) **et al.(2021)** | **Only probiotics keywords** | **48** |
| [**Samani**](https://pubmed.ncbi.nlm.nih.gov/?term=Samani+SA&cauthor_id=36589015) **et al.(2022)** | **Only probiotics keywords** | **49** |
| [**Sadeghirashed**](https://pubmed.ncbi.nlm.nih.gov/?term=Sadeghirashed+S&cauthor_id=34757579) **et al.(2022)** | **Only probiotics keywords** | **50** |
| **Talebi et al.(2022)** | **Only probiotics keywords** | **51** |
| **Wang et al.(2020)** | **Only probiotics keywords** | **52** |
| **Straus et al.(2024)** | **Only probiotics keywords** | **53** |
| **Baiao et al.(2023)** | **Only probiotics keywords** | **54** |
| **Palacios et al.(2020)** | **Only probiotics keywords** | **55** |
| **Nachum et al.(2024)** | **Only probiotics keywords** | **56** |
| **Chan et al.(2022)** | **Only probiotics keywords** | **57** |
| **Leal et al.(2024)** | **Only probiotics keywords** | **58** |
| **Moravejolahkami et al.(2023)** | **Only probiotics keywords** | **59** |
| **Gøbel et al.(2012)** | **Only probiotics keywords** | **60** |
| **Li et al.(2024)** | **Only probiotics keywords** | **61** |
| **Reason: Only probiotics keywords** | | **Total: 61** |
| Authors | **Reason** | **Number** |
| **Cignarella et al.(2018)** | **About gut microbiota and EAE** | **1** |
| **Kim et al.(2021)** | **About gut microbiota and EAE** | **2** |
| **Adamova et al.(2024)** | **About gut microbiota and EAE** | **3** |
| **Katz Sand et al.(2018)** | **About gut microbiota and EAE** | **4** |
| **Stanisavljevic et al.(2016)** | **About gut microbiota and EAE** | **5** |
| **Lee et al.(2010)** | **About gut microbiota and EAE** | **6** |
| **Barone et al.(2021)** | **About gut microbiota and EAE** | **7** |
| **Reason: About gut microbiota and EAE** | | **Total: 7** |
| Authors | **Reason** | **Number** |
| [**Mangalam**](https://www.thelancet.com/journals/ebiom/article/PIIS2352-3964(21)00414-X/fulltext)**et al.(2021)** | **About fungi and protozoa in MS** | **1** |
| [**Aghamohammadi**](https://trialsjournal.biomedcentral.com/articles/10.1186/s13063-019-3454-9#auth-Dawood-Aghamohammadi-Aff1) **et al.(2019)** | **About fungi and protozoa in MS** | **2** |
| **Correale et al.(20110** | **About fungi and protozoa in MS** | **3** |
| **Rosche et al.(2013)** | **About fungi and protozoa in MS** | **4** |
| **Obaid et al.(2022)** | **About fungi and protozoa in MS** | **5** |
| [**Motlagh Asghari**](https://pubmed.ncbi.nlm.nih.gov/?term=%22Asghari%20KM%22%5BAuthor%5D) **et al.(2023)** | **About fungi and protozoa in MS** | **6** |
| **Reason: About fungi and protozoa in MS** | | **Total: 6** |
| Authors | | **Number of study included** |
| **Salehipour et al.(2017)** | | **1** |
| **Dargahi et al.(2020)** | | **2** |
| **He et al.(2019)** | | **3** |
| **Calvo-Barreiro et al.(2020)** | | **4** |
| **Secher et al.(2017)** | | **5** |
| Digehsara et al.(2020**)** | | **6** |
| **Kwon et al.(2013)** | | **7** |
| **Takata et al.(2011)** | | **8** |
| **Rezendea et al.(2013)** | | **9** |
| **Mangalam et al.(2017)** | | **10** |
| **Lavasani et al.(2010)** | | **11** |
| **Salami et al.(2019)** | | **12** |
| **Abdurasulova et al.(2016)** | | **13** |
| **Kouchaki et al.(2017)** | | **14** |
| **Kobayashi et al. (2012)** | | **15** |
| **Ibrahim et al.(2023)** | | **16** |
| **Montgomery et al.(2022)** | | **17** |
| **Chakamian et al.(2023)** | | **18** |
| **Rahimlou et al.(2022)** | | **19** |
| Sadeghi et al. (2022) | | **20** |
| Saisai et al. (2021**)** | | **21** |
| Samani et al. (2022**)** | | **22** |
| Rahimlou et al. (2020) | | **23** |
| **Total:23** | | |
